# Autologous mesenchymal stem cell implantation, hydroxyapatite, bone morphogenetic protein-2, and internal fixation for treating critical-sized defects: a translational study

**DOI:** 10.1007/s00264-019-04307-z

**Published:** 2019-02-12

**Authors:** Ismail Hadisoebroto Dilogo, Phedy Phedy, Erica Kholinne, Yoshi Pratama Djaja, Jessica Fiolin, Yuyus Kusnadi, Nyimas Diana Yulisa

**Affiliations:** 1 Department of Orthopaedics and Traumatology, Cipto Mangunkusumo Hospital, Faculty of Medicine Universitas Indonesia, Jakarta, Indonesia; 2 Stem Cells Medical Technology Integrated Service Unit, Cipto Mangunkusumo Hospital, Faculty of Medicine, Universitas Indonesia, Jakarta, Indonesia; 3 Stem Cells and Tissue Engineering Research Cluster, Indonesian Medical Education and Research Institute (IMERI), Faculty of Medicine Universitas Indonesia, Jakarta, Indonesia; 4 Department of Orthopaedics and Traumatology, Fatmawati General Hospital, Jakarta, Indonesia; 5 Department of Orthopaedics and Traumatology, St. Carolus Hospital, Jakarta, Indonesia; 6 Laboratory of Regenerative and Cellular Therapy (ReGeniC), Bifarma Adiluhung Ltd., Jakarta, Indonesia; 7 Department of Radiology, Cipto Mangunkusumo Hospital, Faculty of Medicine Universitas Indonesia, Jakarta, Indonesia

**Keywords:** Autologous, Mesenchymal stem cell, BMP-2, Critical-sized bone defect, Union

## Abstract

**Introduction:**

Critical-sized defect (CSD) is one of the most challenging cases for orthopaedic surgeons. We aim to explore the therapeutic potential of the combination of bone marrow-derived mesenchymal stem cells (BM-MSCs), hydroxyapatite (HA) granules, bone morphogenetic protein-2 (BMP-2), and internal fixation for treating CSDs.

**Methods:**

This was a translational study performed during the period of January 2012 to 2016. Subjects were patients diagnosed with CSDs who had previously failed surgical attempts. They were treated with the combination of autologous BM-MSCs, HA granules, BMP-2, and mechanical stabilization. Post-operative pain level, functional outcome, defect volume, and radiological healing were evaluated after a minimum follow-up of 12 months.

**Results:**

A total of six subjects were recruited in this study. The pain was significantly reduced in all cases; with the decrease of mean preoperative visual analog scale (VAS) from 4 ± 2.2 to 0 after six month follow-up. Clinical functional outcome percentage increased significantly from 25 ± 13.7 to 70.79 ± 19.5. Radiological healing assessment using Tiedemann score also showed an increase from 0.16 ± 0.4 to 8 ± 3 at one year follow-up. No immunologic nor neoplastic side effects were found.

**Conclusions:**

The combination of autologous BM-MSCs, HA granules, and BMP-2 is safe and remains to be a good option for the definitive treatment for CSD with previous failed surgical attempts. Further studies with a larger sample size are required to be done.

## Introduction

Segmental defects in bone remain an ongoing challenge for orthopaedic surgeons [1]. Large segmental defects, also known as critical-sized defects (CSDs), may not heal spontaneously and lead to nonunion prognosis due to the size of defects or unstable biomechanical properties, unfavourable wound environment, suboptimal surgical technique, metabolic factors, hormones, nutrition, and applied stress [2]. CSDs are difficult to characterize as the diagnosis is subjective [3]. Generally, it has been suggested that the CSD includes defect length greater than 2 cm as well as bone circumference loss greater than 50% [4].

The relative rarity of CSDs means that a high level of evidence to guide their management is sparse [4]. Bone grafts or substitute biomaterials are commonly used as therapeutic strategies for clinical bone surgery to fill the bone defects for reconstructing large bone segments [2]. Over the years, autologous bone grafts (ABGs) have been regarded as the mainstay of therapy to augment or accelerate bone regeneration [5–7]. However, major drawbacks are associated with this approach, such as additional anaesthetic time and personnel needed for graft harvesting [8–10], limited quantity of the graft and access to donor sites, and immune-mediated rejection [5, 8, 11–13].

Hydroxyapatite (HA) is a representative bone repairing biomaterial for its similar composition to human bones and teeth [14]. Compared to ABGs, HA synthetic bone grafts have been shown to stimulate bone regeneration in experimental animal studies, with excellent stability and bone-regenerative characteristics. They slowly degrade and are gradually replaced by bone due to their composition and structure [15].

In addition to ABGs and HA, most surgeons accept that the use of a bone morphogenetic protein (BMP) for treatment of a CSD must be tailored to the individual circumstance. Certainly, the bone involved has a bearing on the acceptable value for a CSD [1]. Previous studies [16–18] have shown that BMP-2 can be utilized in various therapeutic interventions including bone defects and nonunion fractures.

According to the diamond concept of bone healing, as previously described by Giannoudis et al. [19], osteogenic cells must work in conjunction with osteoconductive (scaffold), osteoinductive, and stable mechanical environment. To obtain mechanical stability, fixation is often used for reconstructing bone defects. Most authors believe that solid fixation would facilitate bone union [20]. Compared to internal fixators, external fixators are impractical for patients. Moreover, external fixators need consistent maintenance. Thus, they could be problematic in a population of noncompliant patients [21]. Moreover, external fixation (EF) can lead to numerous complications, including pin-tract infection, joint stiffness, and soft tissue irritation [21]. Thus, we use internal fixation (IF) in this study.

In the present study, we utilized the combination of osteogenic MSCs, osteoconductive synthetic HA granules, and a stable mechanical environment provided by internal fixation to provide a single-stage bone defect reconstruction. This combination had been evaluated in an animal model and significantly resulted in faster and thicker callous formation [22]. To date, human studies evaluating this combination have never been conducted. The purpose of this study is to present the experience of the authors in treating six cases of CSDs using the combination of MSCs, HA granules, BMP-2, and internal fixation in treating CSD.

## Patients and methods

A single arm prospective experimental study was performed between 2012 and 2016. The study protocol was approved by the institutional board review (567/PT02.FK/ETIK/2012) and registered in the clinicaltrials.gov (NCT 01725698). This study was funded by FMUI (Grant Number: 474/H2.F1D/HKP.02.04/2012).

Six patients with CSDs who had failed previous surgical attempts were included. Patients with immunodeficiency, previous history of pathologic fracture, neoplastic pathologies, ongoing hormonal therapy, and suspected active osteomyelitis/related soft tissue infection were excluded. All diagnosis, bone marrow harvest (BMH), and surgical procedures were conducted by one senior trauma consultant orthopaedic surgeon (IHD). Written informed consent and baseline functional status were obtained from all subjects prior to the study period. All subjects were administered with oral ibandronate 150 mg once a month to prevent osteopenia. Characteristics of the subjects are presented in Table 1.

**Table 1:** Patient characteristics

Case	Gender	Age	Affected bone	Duration of disease	Defect volume (cm^3^)	Prev. surgical procedure	Length of follow-up
1	Male	18	Humerus	12 months	5 × 2 × 2 (20 cm^3^)	1. ORIF 2. Implant removal and splinting due to re-fracture	12 months^a^
2	Male	34	Femur	36 months	7 × 3.5 × 3 (73.5 cm^3^)	1. ORIF 2. Debridement, impant removal and external fixation due to infection 3. Debridement, cement spacer	21 months
3	Female	24	Tibia	9 months	12 × 4 × 2 (96 cm^3^)	1. Debridement and ex-fix 2. Debridement and skin graft/flap	17 months
4	Male	28	Tibia	7 years	8 × 3 × 3 (72 cm^3^)	1. Debridement and external fixation 2. Ex-fix removal	12 months
5	Male	33	Tibia	6 months	6 × 2 × 2 (24 cm^3^)	1. Debridement and external fixation	12 months
6	Female	40	Femur	5 years	6 × 2.5 × 2 (30 cm^3^)	1. ORIF 2. Debridement and external fixation 3. Ex-Fix removal	12 months
	Mean ± SD			34.5 ± 31.85	52,58 ± 31.9	19 ± 14.17	

^a^Patient 1 did not continue the follow-up process due to fracture site has consolidated in 6 month

### Isolation and culture of bone marrow-derived mesenchymal stem cells

The autologous BM-MSCs were isolated and cultured based on a protocol previously described by Lubis et al. [23] The BMH was performed under local anaesthesia (lidocaine 2%) in the procedural room of an outpatient clinic in a sterile fashion. Forty milliliters of bone marrow (BM) was aspirated from several locations within the posterior iliac crest and transferred into a container prefilled with 5000 U/mL of heparin. Subsequently, the aspirate was diluted with phosphate-buffered saline on 1:1 ratio and centrifuged at room temperature at 3000 rpm for 30 minutes. The collected buffy coat was washed and transferred into a culture flask containing Dulbecco’s Modified Eagle Medium (Gibco, Grand Island, New York) supplemented with 10% fetal bovine serum (Gibco, Grand Island, New York). Cells were incubated at 37 °C at 5% CO_2_ with routine culture medium change every two to three days. Subculture was performed within seven to ten days.

Attached cells were cultured until they reach at least 50 million cells (4th week). Cellular characterization was subsequently performed on plastic adherent confluent cells by flow cytometry (FACSCalibur™, Franklin Lakes, New Jersey). Cultured cells were checked for typical MSC markers (CD73, CD105) and hematopoietic markers (HLA-DR, CD14, CD19, CD34, and CD45)*.* To ensure safety, the sterility of the BM-MSCs was checked thrice during the culture process. BM-MSC culture procedures were performed in a cGMP-certified facility (ReGeniC Laboratory–Bifarma Adiluhung, Jakarta, Indonesia). Cell viability was evaluated with trypan blue staining using a microscope.

### Surgical procedure

Surgical procedures were performed by one trauma surgeon approximately three weeks after BMH. The nonunion site was exposed; then, fibrotic tissue removal, decortication, and recanalization were performed. Mechanical stability was provided by using an internal fixation system, tailored in accordance with the soft tissue condition. In case 4, the surgery was immediately performed after his external fixation removed (in the same day). The bone defects were filled using HA granules (Bongros®-HA, Bioalpha, Seungnam, Korea) and 1.33 mL BMP-2 (Novosis, CGBio, Seoul, Korea) for each 5 g of HA granule. Prior to the implantation, the scaffolds were mixed with 50 million autologous BM-MSCs contained in 10 mL of plasma solution. Afterward, prompt soft tissue closure was performed.

**Fig. 1:**
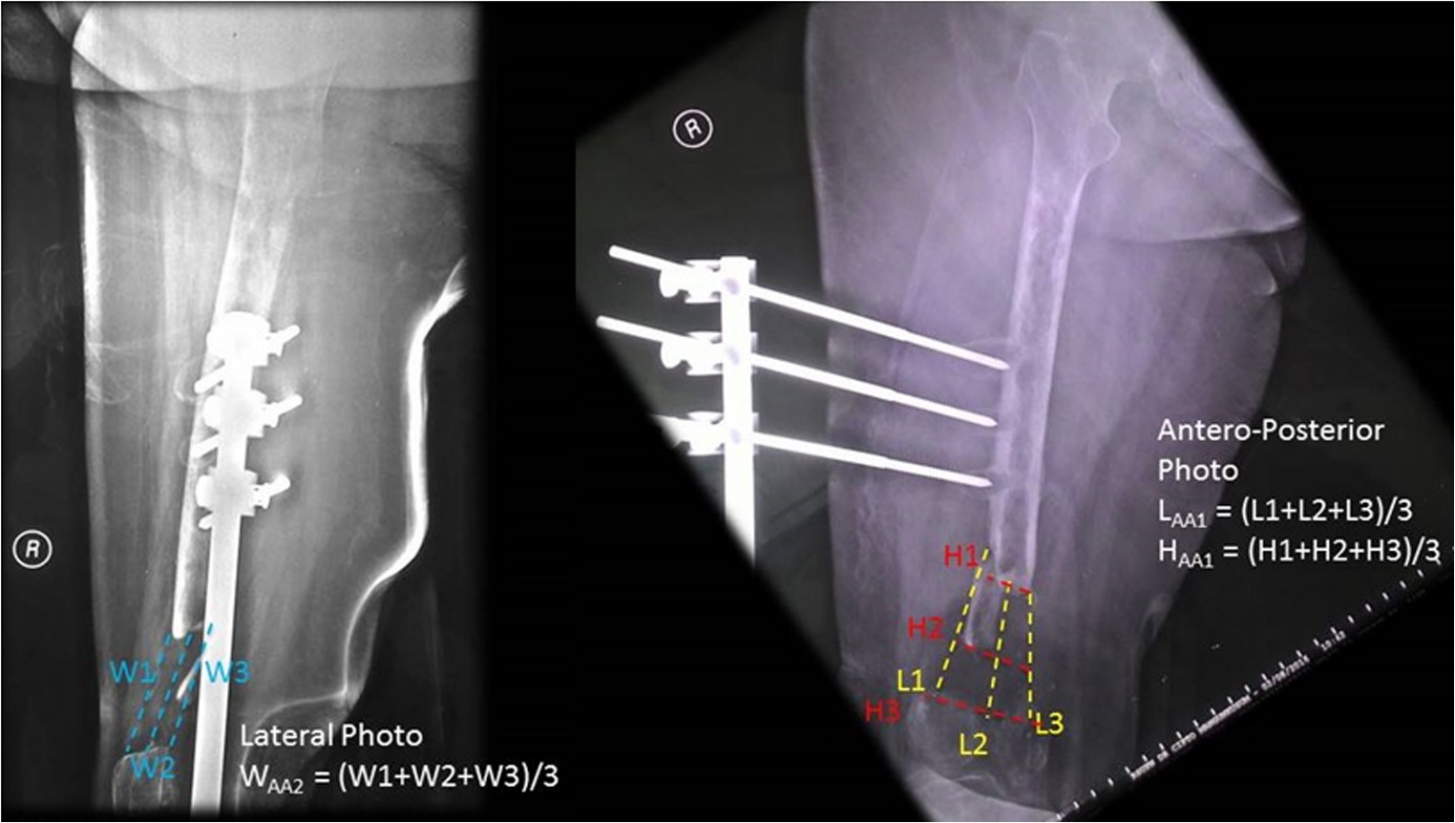
Volume defect measurement by means of radiography

The estimated defect size was measured with the following formula from two projection of plain radiograph of the corresponding extremity as seen in Fig. 1.

$$ V=L\mathrm{AA}1\times H\mathrm{AA}1\times W\mathrm{AA}2 $$

*V*: Volume
*L*: Length
*H*: Height
*W*: Width

Bone defect volume was reported in cm^3^. For instance, if the defect volume is 30 cm^3^, six vials of HA granules (Bongros®-HA) at 5 g (total 6 × 5 = 30 g) will be filled into the defect.

### Evaluation of the subjects

Subjects were hospitalized for five days after surgery. The initial clinical evaluation was performed for signs of pain, infection, and soft tissue compromise. In lower extremity cases, no weight bearing was allowed for at least six weeks after surgery. Patients underwent clinical and radiographic evaluation every month. The measured outcomes were pain level (VAS) and functional scores of the involved extremity, which was scored based on either Lower Extremity Functional Scale [24] (LEFS) or Disabilities of the Arms, Shoulder, and Hand [25] (DASH).

Radiological assessments were conducted using Tiedeman radiological scoring system for measuring volume defect before as well as six and 12 months after implantation. However, there is no consensus regarding the cumulative score for radiological union. We determined that Tiedeman score ≥ 5 to be the cut-off point for radiographic union [26].

## Results

In all subjects, no major complication occurred intra-operatively or during the post-operative period. However, two subjects (case 2 and case 4) developed surgical site infection. Case 2, a 34-year-old male, after undergoing spacer removal, open reduction internal fixation and autogenic BM-MSC implantation, developed surgical site infection. Subsequently, he underwent re-debridement and re-implantation of allogeneic umbilical cord MSC therapy, and his infection resolved. Case 4, a 28-year-old male, had superficial surgical site infection. After we performed wound care and dressing as well as administered antibiotics, the infection resolved. Case 6, a 40-year-old female, developed partial union. We planned to administer another MSC implantation for case 6, but she did not do follow-up to our hospital as she had family problems and moved to another island.

**Fig. 2:**
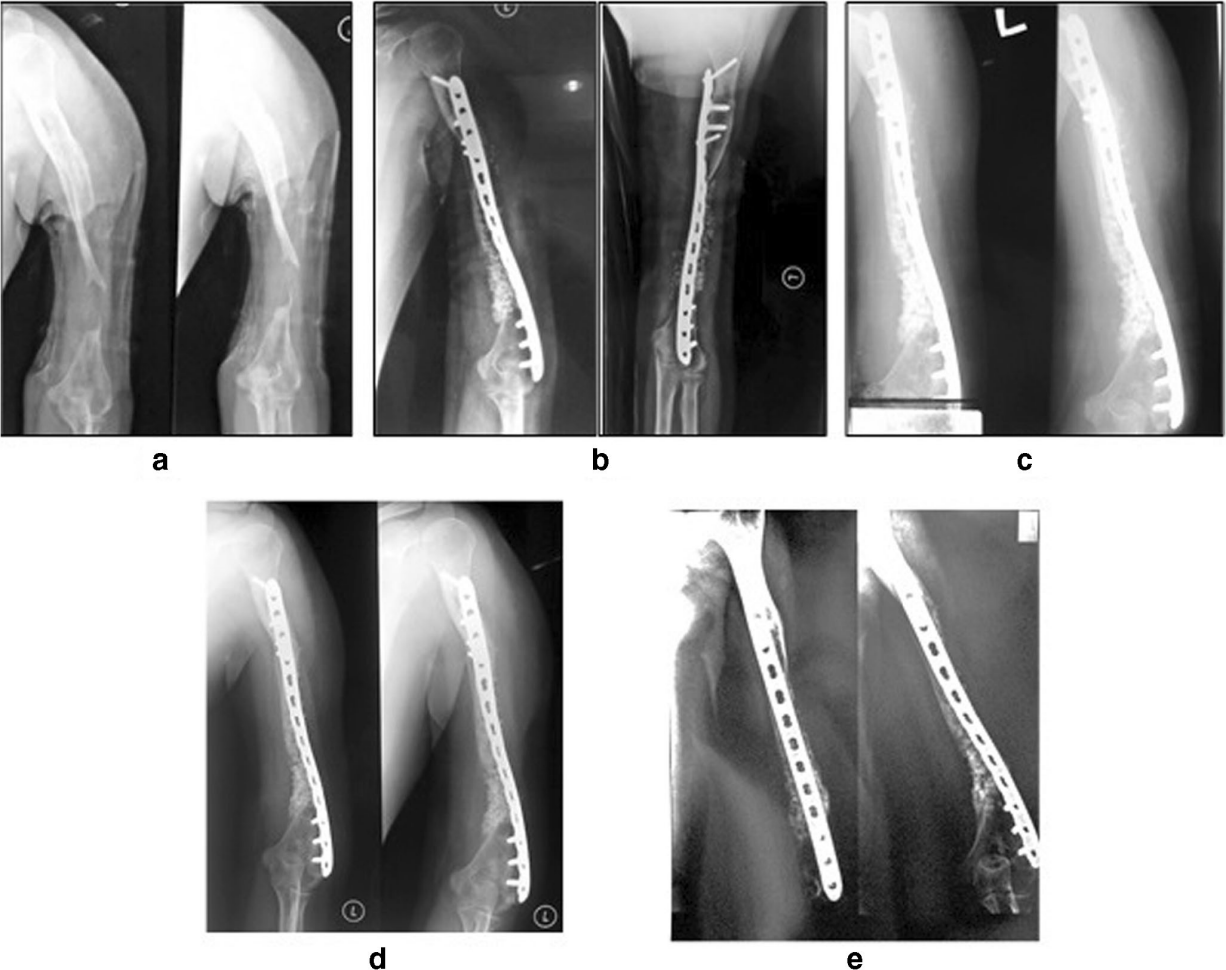
An 18-year-old male (case 1) with 5-cm bone defect of the humerus. **a** Pre-operative radiography. **b** Post-operative radiography. **c** Two-month post-operative radiography. **d** Six-month post-operative radiography. **e** Twelve-month post-operative radiography

**Fig. 3:**
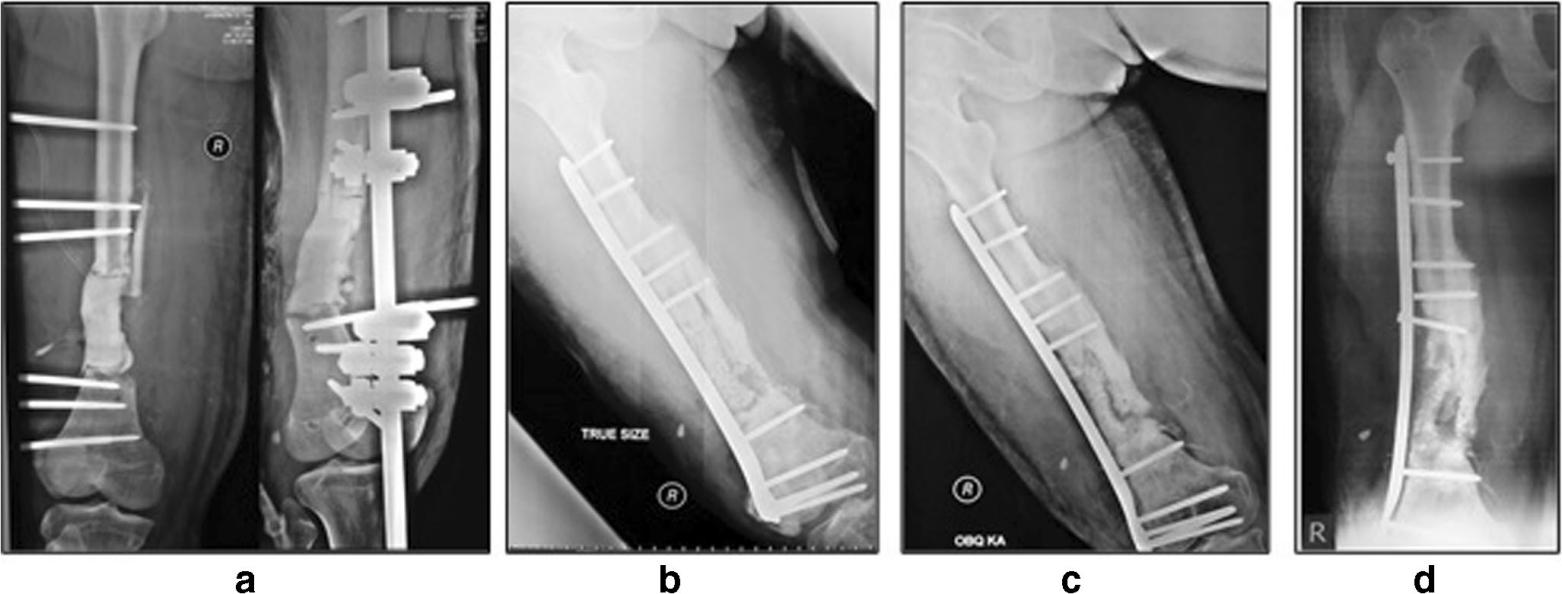
A 34-year-old male (case 2) diagnosed with infected open fracture of the right distal femur with 7-cm bone defect after Masquelet procedure. **a** Pre-operative radiography. **b** Post-operative radiography, **c** Twelve-month radiography. **d** Twenty-one-month radiography

**Fig. 4:**
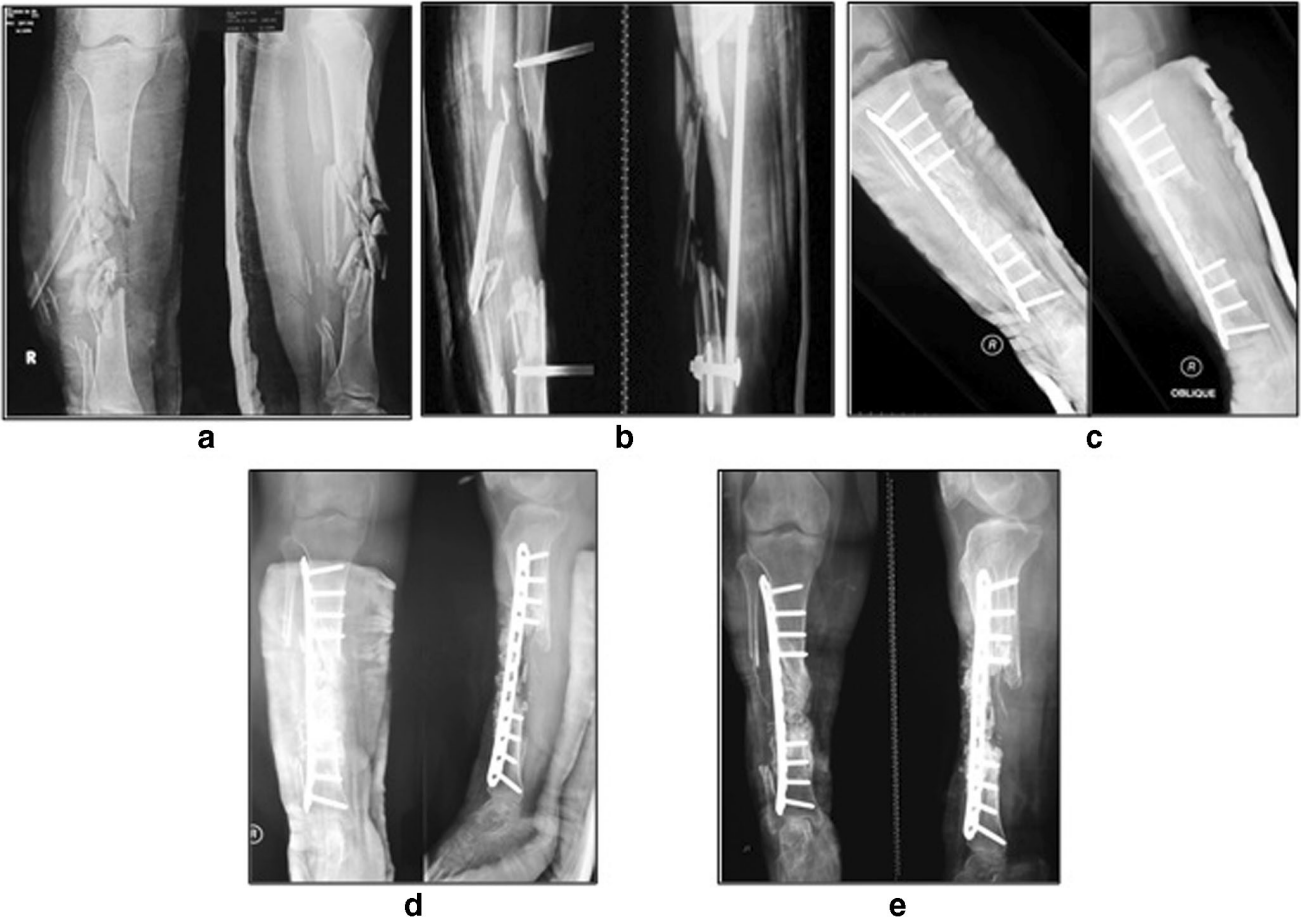
A 24-year-old female (case 3) with 12 cm BD of the tibia. **a** Initial radiography. **b** Pre-operative radiography. **c** Post-operative radiography. **d** Six-month radiography. **e** Fifteen-month post-operative radiography

**Fig. 5:**
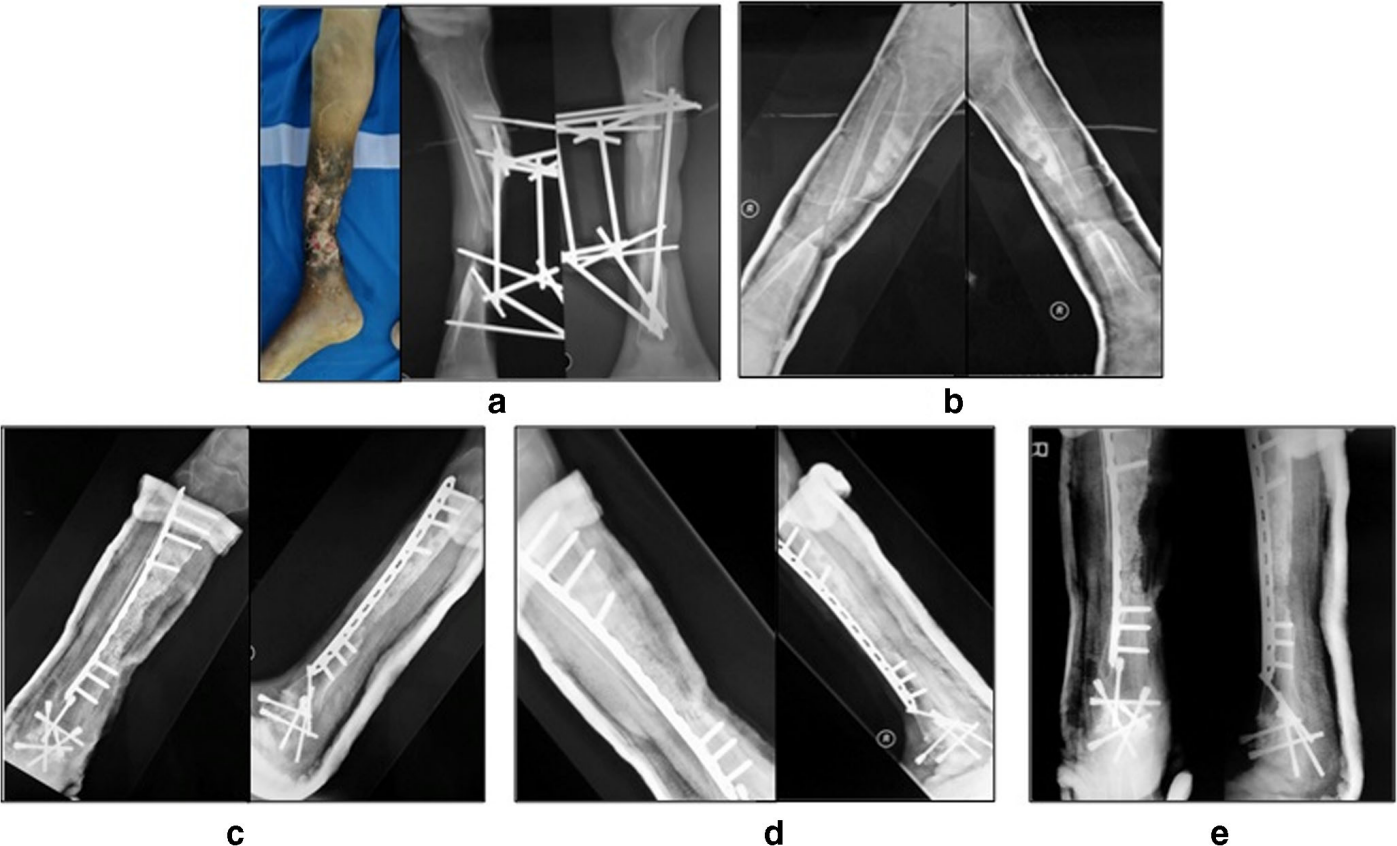
A 28-year-old male (case 4) with 7-year history of 8-cm bone defect of the right tibia. **a** Initial x-ray and clinical picture after external fixation removal. **b** Pre-operative radiography. **c** Post-operative, **d** Five-month radiography. **e** Twelve-month radiography

**Fig. 6:**
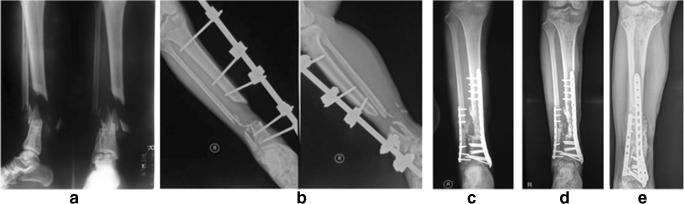
A 33-year-old male (case 5) with 6-cm bone defect of the tibia. **a** Initial radiography. **b** Pre-operative radiography. **c** Post-operative radiography. **d** Six-month post-operative radiography. **e** Twelve-month post-operative radiography

**Fig. 7:**
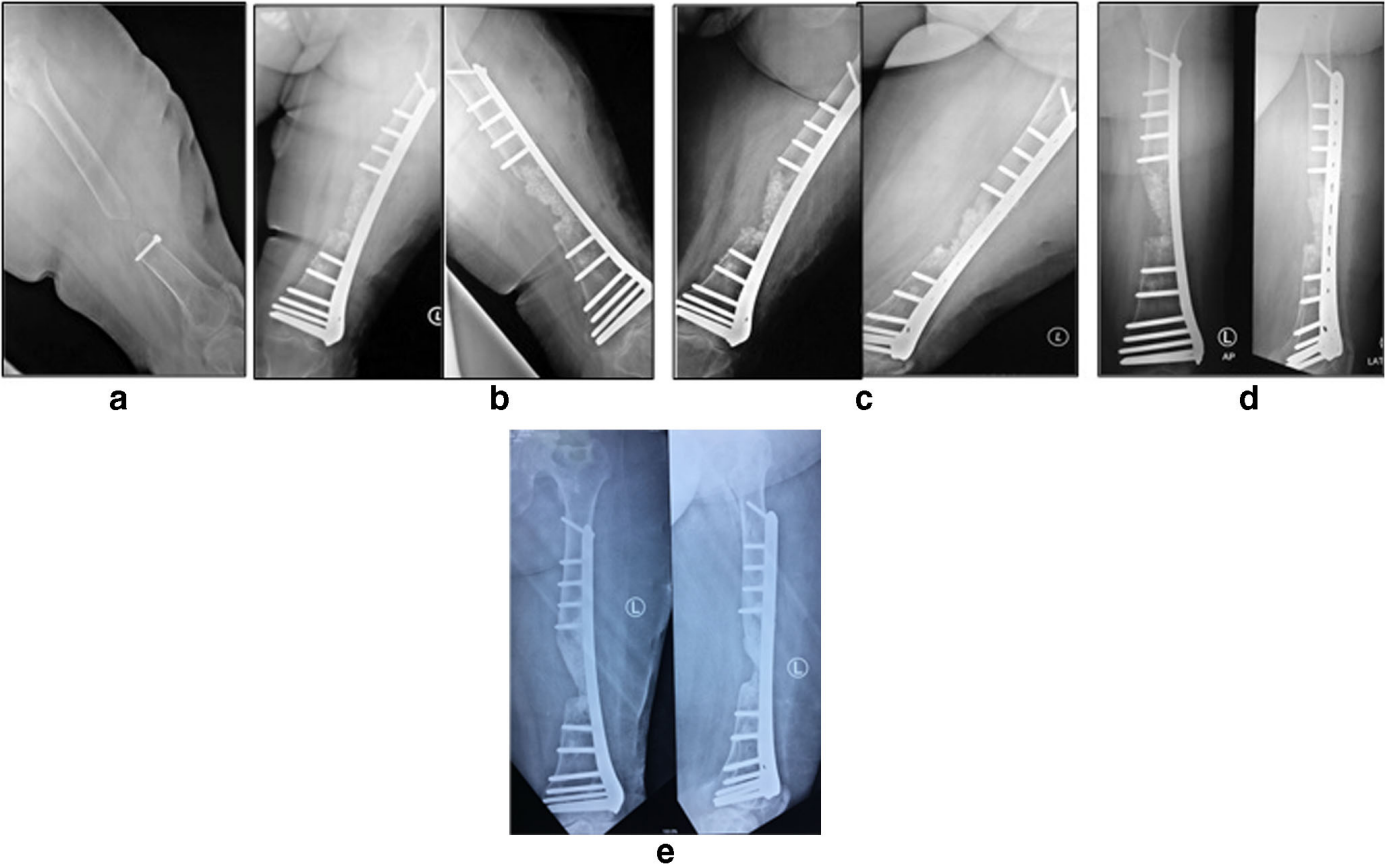
A 40-year-old female with 5 years of 6-cm bone defect of the femur. **a** Pre-operative radiography. **b** Post-operative radiography. **c** Six-month post-operative radiography. **d** Twelve-month post-operative radiography. **e** Four-year post-operative radiography

All cases were regularly followed until union was achieved with a mean duration of follow-up of 19 ± 14.17 months. The initial mean longitudinal bone defect measured 7.33 cm (range, 5–12 cm) or 52.58 ± 31.9 cm^3^ in volume bone defect. The outcome comparison between pre-operative, after 6-month, and 12-month follow-up is presented in Tables 2 and 3. Pre-operative, post-operative, and follow-up radiograph is shown in Figs. 2, 3, 4, 5, 6, and 7 showing dramatic improvement of the bone graft incorporation through times.

**Table 2:** The effect of MSCs implantation for critical-sized bone defect on primary end point outcomes

	Pre-op (*n* = 6)	6 month post-op (*n* = 6)	12 months post-op (*n* = 5)
Visual analog scale	4 ± 2.2	0	0
Functional score (%)	25 ± 13.7	71.59 ± 20.5	78.23 ± 15.4
Defect volume (cm^3^)	52.58 ± 31.9	2.83 ± 3.54	3.4 ± 3.65
Tiedeman score	0.16 ± 0.4	8 ± 3	9 ± 2

**Table 3:** Functional score and leg length discprepancy of the subjects

Case	DASH	LEFS	LLD
1	25 (initial) 20.83 (3-month-post-operative) 16.7 (6-month-postoperative) 12.5 (9-month-postoperative)- 10 (1-year-post-operative)	–	–
2	–	–	2.5 cm (initial), 1 cm (2-month-post-operative)
3	–	26.25% (initial), 55% (3-month-post-operative), 60% (6-month-post-operative), 72.5% (9-month-post-operative), 82.5% (12-month-post-operative)	3 cm (initial), 1 cm (3-month-post-operative)
4	–	22.5% (initial), 55% (3-month-post-operative), 62.5% (6-month-post-operative), 75% (9-month-post-operative), 80% (12-month-post-operative)	3 cm (initial), 3 cm (14-month-post-operative)
5	–	22.5% (initial), 43.75% (1-month-post-operative), 62.5% (3-month-post-operative), 80% (6-month-post-operative), 100% (9- and 12-month-post-operative)	3 cm (initial), 0 cm (12 months post-operative)
6	–	25% (initial), 38.75% (1-month-post-operative), 52.5 (3-month-post-operative), 60% (6-month-post-operative), 70% (12-months post-operative)	5 cm (initial), 0.5 cm (6-month-post-operative)

## Discussion

The major issue in CSDs aside from the size of the defect itself is the limited capabilities of the biological environment to promote fracture healing. The diamond concept of fracture healing describes that osteogenic cells, growth factors (osteoinduction), osteoconductive scaffolds, and mechanical environment are the cornerstone of fracture healing [19, 27]. During the early phase of healing, haematoma is the source of signaling molecules that may induce a cascade of cellular events that initiate the fracture healing process. Stimulated by these growth factors, the MSCs are recruited to the fracture site and transform to osteoblasts to promote further healing. Osteoconductive materials provide natural scaffold for all the aforementioned cellular events stabilized mechanically to optimize the healing process [19]. Various treatments are available for treating CSDs; however, all of them revolve around this concept.

In the present study, we applied the basis of this concept by using the combination of MSCs, BMP-2, HA scaffold, and IF. To our knowledge, this is the largest series that evaluates the application of MSC-based tissue engineering construct in CSDs. To evaluate the effectiveness of this technique, we selected a group of “very challenging” cases—those who had poor results on the previous reconstructive procedures.

Masquelet technique is the current recommended treatment for CSDs with a high rate of success [3]. However, some authors have documented a small number of failures using this technique. The main cause of failure was reactivation of infection, graft reabsorption, and graft maturation failure [28, 29]. The technique was performed on the second case by giving an antibiotic cemented spacer in the defect by the previous surgeon. However, during the definitive surgery, no membranes were found on the cemented region. Thus, the patient was referred to our institution for further treatment.

One of the most challenging cases was case 5. The patient had extensive soft tissue damage around the fracture site that warranted a soft tissue reconstruction in the previous institution despite the presence of a massive bone defect. The quality of soft tissue around the defect would complicate whatever type of reconstruction that we would choose, not to mention the initial longitudinal defect was 12 cm. The combination of HA, MSCs, BMP-2, and mechanical stability provided by lateral side plating has achieved a complete consolidation after nine months. In addition, the patient had significant functional improvement in the last follow-up.

During the past decades, numerous studies documented a wide number of varieties in tissue engineering constructs in orthopaedic field, especially in treating tibia fractures, nonunion, and CSDs [30–32]. Several types of osteogenic cells have been applied in tissue engineering so far, such as aspirate, concentrated aspirate, periosteum-derived MSCs, and in vitro expanded MSCs [30]. However, despite the early enthusiasm of the promising result in the animal studies, clinical studies that applying them in treating large bone defect are scant.

Quarto et al. [32] and Marcacci et al. [31] applied the in vitro expanded MSCs for treating 4–7 cm bone defect in six patients. The bone defect was supported by the use of porous hydroxyapatite-tri-calcium-phosphate (HA-TCP) scaffolds that was designed to match the size and shape of the defect. Complete fusion and integration of the scaffold and host bone were achieved at five to seven months, showing promising results in the repairment of CSD. Compared to HA construct, HA-TCP construct demonstrated superiority regarding cell proliferation, calcium deposition, and collagen bundle formation [33, 34].

Bajada et al. [35] successfully treated a nine year tibial nonunion resistant to six previous surgical procedures by using autologous bone marrow stromal cells expanded to 5 × 10^6^ cells after three week tissue culture. The cells were combined with calcium sulfate (CaSO4). However, despite its success, calcium sulfate’s low biomechanical performance and rapid resorption were not considered to be a suitable scaffold in CSDs, in which the biomechanical property of the scaffold is also needed to permit osteoconduction [36]. Particle size is also an important variable in bone regeneration. Malinin et al. [37] have found that particle size between 100 and 300 μm is the best option for impaction grafting in closed intraosseous bone defects. However, as impaction grafting is not possible in most CSDs, innate mechanical strength of the allograft should also be noticed, Sanchez et al. [38] have suggested that intermediate particle size (300–600 μm) has best mechanical strength due to particle morphology and number of bonds in the contact zone.

According to previous studies, aspirated BM contained an average of 600–700 cells/cm^3^. Our cell and tissue culture process had expanded these numbers of cells into 50 × 10^6^ cells during three replicative passages in the span of three weeks. Hernigou et al. [39] reported that the use of percutaneous ABGs was effective and safe for nonunion. Moreover, they mentioned that the efficacy of the ABGs appeared to be in proportion with the concentration and total amount of cells injected to the graft.

Osteoinductivity plays a vital role in the regeneration of CSDs. It is considered as the possible cause why inconsistent results were found with the use of earlier tissue-engineering construct involving MSCs and resorbable calcium scaffold [40]. Niikura et al. [41] revealed that downregulation of BMP gene expression might account for nonunions; this suggests that BMPs play an essential role in osteogenesis. The beneficial effect of BMP-2 itself has been approved by the FDA for treating nonunions [16]. The osteoinductive activities of BMPs have led to numerous applications in bone regeneration. However, the introduction of BMP products to the market was not without reports of multiple complications and adverse effects. Efforts have been focused on improving the delivery of BMPs to lower the administration dosage and maintaining its local concentration to lengthen its duration of action [42]. Few strategies have been proposed to overcome these issues. Several gene therapy studies have successfully transferred the BMP-2 cDNA to the muscle grafts or local tissue to induce repair of segmental bone grafts but still limited in an animal model [18, 43]. As for clinical studies, Carragee et al. [44] reviewed the efficacy and safety of rhBMP-2 treatments in spinal surgery. Although the original report from industry-sponsored rhBMP-2 publications found no adverse events associated with the use of rhBMP-2, the review of FDA documents and subsequent publications proven otherwise. The estimated adverse events associated with the use of rhBMP-2 range from 10 to 50%. Several serious side effects of BMP-2, including heterotopic ossification, early osteolysis, and inflammation at the grafting site were also reported [41].

A recent animal study by Decambron et al. evaluated the impact of adding BMP-2 to MSCs-coral tissue engineering construct (TEC). Despite insignificant results, an increased amount of newly formed bone and scaffold resorption was observed in BMP-MSC-TEC group [40]. The contradictory effects of the BMP on bone healing are explained by its stimulating effect to both osteoclasts and osteoblasts. In the presence of both cells, BMP will primarily activate the osteoclasts to promote bone resorption supporting osteoclastogenesis [45].

In several conditions, including being in a poorly vascularized environment, state of extensive tissue damage and inadequate nutrition transplanted stem cells cannot be predicted and would fail as a treatment. MSCs cannot work alone; they depend on the extracellular matrix, and they should be combined with an intercellular signal (osteoinductive) as well as neovascularization to sustain its viability. The BMP-2 has the strongest osteoinductive potency compared to other osteoinductive factors. It plays an essential role in the process of differentiation which starts from the differentiation of MSC into osteoprogenitor cells and subsequently become pre-osteoblast, and eventually the differentiation of osteoblast into osteocyte [46].

The findings in our study showed a promising result in promoting fracture healing and scaffold integration in the host bone. MSC-based tissue engineering construct may also avoid some drawbacks that are experienced by other treatment methods such as the limited number of graft/scaffold, requirement of multiple surgery or microvascular surgery, and long treatment duration that extremely relied on the patient compliance. There are several limitations in our study. The absence of control group may largely affect the results and conclusions of our study, as we could not evaluate the efficacy of each component separately. Our study only involved a small number of patients. These limitations highlighted that further studies, multicenter randomized controlled studies with larger samples, and longer follow-up duration are required to follow our translational study.

Previously, we published a separated case report on a similar technique for reconstructing CSDs after osteofibrous dysplasia resection. The patient obtained complete union and significant functional improvements by 42 weeks, and the results were consistent after 84 weeks of follow-up [46]. This study further supports our in vitro study that the additional MSC injection to the scaffold would increase the potency of bone healing [27]. Aside from their effects on stimulation of the dormant local stem cells surrounding the fracture site by its paracrine effects, the induction of proliferation and proliferation of BM-MSCs into osteogenic cells (when implanted in the defect area) is more desired in CSDs.^47^

## Conclusions

The combination of autologous BM-MSCs, HA granules, and BMP-2 are safe and may serve as a good option for treating CSDs. Further multicenter randomized controlled trials are required to investigate the efficacy of this therapeutic combination.
